# Mental health outcomes among United States remote-eligible working adults post COVID-19: national evidence

**DOI:** 10.3389/fpubh.2026.1809511

**Published:** 2026-04-28

**Authors:** Muinat Abolore Idris, Jingjing Gao, Juan Aguilera, Louis D. Brown

**Affiliations:** 1 Department of Environmental and Occupational Health Sciences, School of Public Health, The University of Texas Health Science Center at Houston, Houston, TX, United States; 2 Department of Management, Policy and Community Health, School of Public Health, The University of Texas Health Science Center at Houston, Houston, TX, United States; 3 Department of Health Promotion and Behavioral Sciences, School of Public Health, The University of Texas Health Science Center at Houston, Houston, TX, United States

**Keywords:** anxiety, depression, disorder diagnosis, health disparities, medication use, mental health, remote work, remote-eligible working adults

## Abstract

**Background:**

The COVID-19 pandemic accelerated the transition from traditional office-based work to remote work, but the national-level relationship between remote work and workers’ mental health remains understudied.

**Objective:**

To assess the prevalence of six mental-related health outcomes among remote-eligible working adults in the United States and examine sociodemographic and health-related disparities.

**Methods:**

The 2023 National Health Interview Survey (NHIS) adult sample data were analyzed for remote-eligible (*n* = 5,813) and non-remote-eligible occupations (*n* = 11,644). Participants’ self-reported depression and anxiety frequency, depression and anxiety disorder, and depression and anxiety medication use. Survey-weighted ordered logistic regression and cumulative logistic regression models were used to estimate interaction effects, and potential confounders were adjusted for.

**Results:**

Mental health outcomes among remote-eligible- working adults varied substantially across sociodemographic groups, with obvious gaps between depression/anxiety frequency, symptom burden, and depression/anxiety disorders diagnosis, particularly among males, older adults, and non-Hispanic Asian remote-eligible working adults.

**Conclusion:**

With the projected aging workforce, the need for targeted screening, equitable and culturally responsive mental health care, and equitable mental health care services to address unmet mental health needs is crucial with the expanding remote-work structure.

## Background

The COVID-19 pandemic facilitated the global shift toward remote work, changing the traditional office workplace models to remote work, and urging inquiry into the potential long-term impact of working remotely on mental health. While working remotely offers well obvious advantages, including reduced commuting time, increased flexibility, and autonomy, it also introduces new challenges, including social isolation, reduced face-to-face interactions, blurred work-life boundaries, and unequal resources and technology access (1–3). These challenges may significantly impact psychological well-being, leading to mental health issues, as studies have shown the complex and often mixed mental health impacts of working remotely. For instance, Kitagawa et al. reported that working from home (remotely) improves workers’ sleep quality, likely due to reduced commute time and lower alcohol consumption, which may positively contribute to good mental health. Studies also show that while some workers benefit from improved work-life balance and reduced physical health risks (4, 5) from working remotely, some experience increased stress, anxiety, and burnout due to social isolation and lack of spontaneous peer support (6, 7). However, considering the structure of workers’ home environment, these effects may not be uniform due to individual worker coping resources, organizational context, job type, and living arrangements, which may influence worker behavior and impact psychological well-being. For example, Covelli et al. (6) found that social service workers who worked remotely during the COVID-19 pandemic experienced higher anxiety levels compared to their in-person counterparts. Similarly, healthcare workers, particularly those lacking social support or living alone, experienced increased insomnia, anxiety, and depression rates during COVID-19 (8–10). While these experiences are not unique among healthcare workers, the conditions could have been exacerbated by COVID-19. In China, both during and after the pandemic, frontline healthcare workers had elevated levels of anxiety and depression (11, 12)—the two main indicators of psychological distress that contribute to poor mental health.

Beyond healthcare workers, the United States general population has also experienced increased psychological distress arising from prolonged social isolation, work overload, and the associated broader disruptions with COVID-19. These shifts have made it increasingly important to understand how post--pandemic work environments—particularly the rapid expansion of remote work—shape mental health among employed adults. Emerging evidence suggests that remote work may act as a social determinant of mental health. Although a recent analysis of the United States. Household Pulse Survey data found that individuals working remotely 1 to 4 days per week had slightly lower odds of depression risk, whereas- those working remotely five or more days per week had moderately higher odds of anxiety risk compared with non-remote workers (13). Notably, this study did not examine how sociodemographic or health-related covariates influence these associations, highlighting an important gap in the literature.

Despite growing attention to remote work and mental health, few studies have comprehensively assessed multiple mental health outcomes, including symptom frequency, diagnosis, and medication use among remote eligible working adults. Prior studies have also rarely examined how health indicators and sociodemographic characteristics shape these patterns, limiting the understanding of the potential disparities in mental health care diagnosis and treatment. To address these, we aims to: (i) examine the pattern and prevalence of depression and anxiety symptom, the diagnosed disorders, and medication use among United States remote-eligible working adults; and (ii) evaluate sociodemographic and health indicators associated with these outcomes, at the national level to inform occupational health practice and guide targeted mental health interventions, specifically for remote-eligible working adults.

## 2 Methods

### 2.1 Study design and data source

This study used the 2023 National Health Interview Survey (NHIS) Adult Sample dataset, an annual, national representative survey administered by the National Center for Health Statistics (NCHS) that collects comprehensive information on health status, healthcare access, and health-related behaviors of the civilian, noninstitutionalized United States population. The survey employed a complex, multistage probability sampling design to ensure national representativeness.

**Figure 1:**
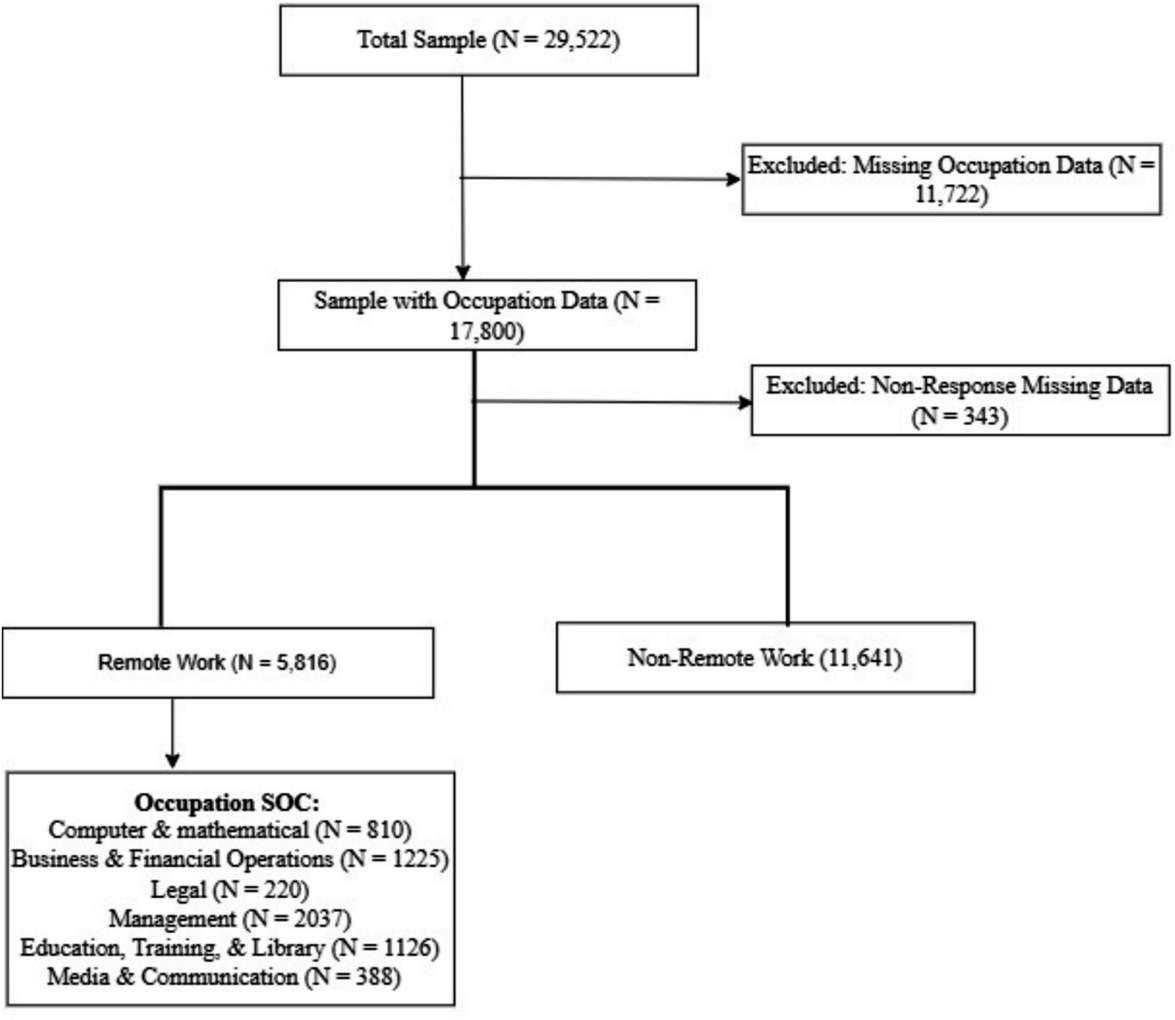
Flowchart of final analytic sample selection.

The Adult Sample data include 29,522 participants; of these, we analyzed 17,457 and excluded 12,065 due to missing occupation codes or responses (Figure 1). Participants’ occupations were accessed from the “EMDOCCUPN2_A” variable. We accessed participants’ occupational information rather than industry, to ensure representativeness, given greater variability in remote work feasibility across occupations. Participants were considered remote-eligible working adults if their occupation fall under the following six primary Standard Occupational Classification (SOC) codes: “SOC 15–0000 (Computer and Mathematical Occupations),” “SOC 13–0000 (Business and Financial Operations),” “SOC 23–0000 (Legal Occupations),” “SOC 11–0000 (Management Occupations),” “SOC 25–0000 (Education, Training, and Library Occupations),” and “SOC 27–0000 (Media and Communication Occupations)” (Figure 1). While NHIS does not collect data or categorize participants by remote work status, we inferred the classification using occupational categories identified by the Bureau of Labor Statistics (BLS) through the Current Population Survey (CPS) (14). The CPS assessed whether people worked from home for pay and, if so, for how many hours, which was used to define “telework,” which we referred to as remote-eligible. This classification reflects only the potential for working adults to be considered remote-eligible (i.e., remote-eligible working adults) based on occupation, per the SOC codes, rather than directly measuring whether participants worked remotely. This approach is further supported by the methodology outlined in Dingel and Neiman’s (15) study and has also been used by Nargis et al. (16).

### 2.2 Measured variables

#### 2.2.1 Mental health outcomes

Information on six mental health outcomes—anxiety frequency, anxiety disorder, anxiety medication use, depression frequency, depression disorder, and depression medication use—was obtained from participant responses to the following questions: (1) “How often do you feel worried, nervous, or anxious?” (2) “Have you EVER been told by a doctor or other health professional that you had any type of anxiety disorder?” (3) “Do you take prescription medication for these feelings (anxiety)?” (4) “How often do you feel depressed?” (5) “Have you EVER been told by a doctor or other health professional that you had any type of depression?” (6) “Do you take prescription medication for depression?” Anxiety and depression frequency were reported on a five-point ordinal scale and were recoded, ranging from 0 (“never”) to 4 (“daily”) to preserve the ordinal structure. Responses to anxiety and depression disorder diagnosis and medication use were binary (yes/no). Participants who responded “Yes” to the anxiety disorder and depression disorder diagnoses were classified as having anxiety disorder and depression disorder, respectively, and those reporting prescription use were classified as using anxiety medication or using depression medication. Responses coded as refused, not ascertained, or do not know were excluded from the analysis.

#### Covariates

Self-reported sociodemographic and health indicator characteristics, including age, sex, education, race/ethnicity, marital status, health insurance, body mass index (BMI), and general health status, were included as categorical variables. The covariate, marital status, was derived from the question: “Are you now married, living with a partner together as an unmarried couple, or neither?,” which was recoded as 1 = married and 0 = not married (living with a partner or neither). Responses with missing or invalid values were excluded.

### Statistical analysis

Descriptive characteristics of participants were compared by remote-eligible work status using a design-based Pearson Chi-square test. After adjusting for all covariates, associations among the six mental health outcomes were examined using survey-weighted ordinal logistic regression models. Adjusted odds ratios (aORs) and 95% confidence intervals (95% CIs) were reported. Statistical significance was evaluated using two-sided tests, with *p*-values <0.05, <0.01, <0.001, and <0.0001. To account for the complex NHIS survey design, all analyses incorporated sampling weights, clustering, and stratification. Analyses were conducted in SAS version 9.4 (SAS Institute Inc., Cary, NC).

### Ethical considerations

This study is exempt from the UTHealth Houston IRB because a de-identified, publicly available secondary dataset was used. All analyses complied with the data use guidelines provided by the NCHS and conformed to ethical principles outlined in the Declaration of Helsinki.

## Results

Table 1 presents weighted descriptive statistics comparing remote and non-remote eligible workers. On average, United States adults who work remotely are mostly non-Hispanic White (68.7% vs. 57.6%, *p* < 0.0001), married (61.9% vs. 46.2%, *p* < 0.0001), hold higher education degrees beyond some college degrees (*p* < 0.0001), and have health insurance (96.2% vs. 89.8%, *p* < 0.0001).

**Table 1:** Weighted descriptive characteristics of remote-eligible and non-remote-eligible working United States Adults.

Variable	Category	Non-remote (%)	Remote (%)	*p*-value
Sociodemographic characteristics				
Sex	Female	46.7	47.2	0.54
	Male	53.3	52.8	
Age group (yrs)	18–24	17.8	7.3	<0.0001
	25–34	21.8	22.6	
	35–44	19.5	24.4	
	45–54	17.9	21.0	
	55–64	15.4	17.2	
	65 and older	7.6	7.5	
Race/Ethnicity	Hispanic	21.7	11.0	<0.0001
	Non-hispanic white	57.6	68.4	
	Non-hispanic black	12.6	8.9	
	Non-hispanic Asian	5.4	9.1	
	Others	2.7	2.6	
Insurance status	Insured	89.8	96.2	<0.0001
	Uninsured	10.1	3.8	
Marital status	married	46.2	61.9	<0.0001
	Not married	53.8	38.1	
Education	<High school	10.1	1.4	<0.0001
	High school	31.3	10.6	
	Some college/AA	33.7	21.7	
	Bachelor’s degree	17.0	37.8	
	Graduate degree	8.0	28.5	
Health indicators				
BMI	Underweight	1.4	1.2	0.003
	Normal	29.5	30.8	
	Overweight	33.6	35.7	
	Obese	35.5	32.3	
Health status	Excellent	24.6	27.4	<0.0001
	Very good	36.1	40.9	
	Good	29.1	25.5	
	Fair	9.2	5.5	
	Poor	1.0	0.8	
Mental health outcomes				
Anxiety frequency	Never	27.9	20.7	<0.0001
	A few times a year	29.0	32.4	
	Weekly	16.8	19.0	
	Monthly	12.5	15.0	
	Daily	13.8	12.9	
Anxiety disorder	No	82.0	82.9	0.217
	Yes	18.0	17.1	
Anxiety medication	No	87.4	86.6	0.264
	Yes	12.6	13.4	
Depression frequency	Never	52.8	51.1	<0.0001
	A few times a year	28.4	33.2	
	Monthly	9.1	8.8	
	Weekly	6.4	4.7	
	Daily	3.3	2.3	
Depression disorder	No	82.0	84.0	0.006
	Yes	18.0	16.0	
Depression medication	No	90.2	90.4	0.843
	Yes	9.8	9.6	

Tables 2, 3 present the survey-weighted ordinal logistic regression analyses for depression and anxiety, respectively, with the full adjusted models for remote-eligible adults in the United States.

**Table 2:** Survey-weighted ordered logistic regression analysis of United State Remote-eligible adults by depression frequency, depression disorder, and depression medication usage post COVID-19.

Variable	Depression frequency [aOR (95% CI)]	*p*-value	Depression disorder [aOR (95% CI)]	*p*-value	Depression medication [aOR (95% CI)]	*p*-value
Occupation [Remote-eligible] (Ref: others)	0.99 (0.91–1.06)	0.71	0.86 (0.77–0.97)	0.014	0.87 (0.76–1.00)	0.05
Sociodemographic characteristics						
Sex (Ref: Female)		<0.0001		<0.0001		<0.0001
Male	1.66 (1.55–1.78)		0.42 (0.38–0.46)		0.36 (0.32–0.41)	
Age group (Yrs) (Ref: 18–24)		<0.0001		<0.0001		0.002
25–34	1.12 (0.97–1.29)		0.99 (0.82–1.21)		0.96 (0.75–1.24)	
35–44	1.37 (1.19–1.58)		0.81 (0.67–0.98)		1.00 (0.78–1.28)	
45–54	1.88 (1.62–2.17)		0.60 (0.48–0.74)		0.80 (0.62–1.05)	
55–64	2.18 (1.86–2.55)		0.46 (0.38–0.57)		0.76 (0.59–0.98)	
65 and older	2.92 (2.47–3.46)		0.42 (0.34–0.54)		0.70 (0.53–0.93)	
Race/ethnicity (Ref: hispanic)		<0.0001		<0.0001		<0.0001
Non-hispanic (NH) white	0.55 (0.49–0.62)		2.38 (2.04–2.78)		2.85 (2.29–3.54)	
NH black	0.84 (0.72–0.97)		1.06 (0.85–1.33)		1.04 (0.78–1.38)	
NH Asian	0.95 (0.79–1.13)		0.50 (0.36–0.69)		0.42 (0.27–0.65)	
Others	0.58 (0.44–0.76)		2.20 (1.59–3.06)		1.85 (1.19–2.88)	
Insurance status (Ref: Uninsured)		0.592		0.05		0.0001
Insured	1.04 (0.90–1.20)		1.22 (0.99–1.49)		1.87 (1.36–2.59)	
Marital Status (Ref: not married)		<0.0001		<0.0001		<0.0001
Married	1.55 (1.45–1.66)		0.67 (0.60–0.74)		0.67 (0.59–0.77)	
Education (Ref: <High School)		<0.0001		<0.0001		<0.0001
High School	0.92 (0.77–1.09)		1.14 (0.89–1.47)		1.86 (1.24–2.81)	
Some College/AA	0.74 (0.62–0.87)		1.47 (1.15–1.88)		2.44 (1.63–3.65)	
Bachelor’s Degree	0.69 (0.58–0.82)		1.51 (1.16–1.97)		2.65 (1.74–4.02)	
Graduate Degree	0.67 (0.55–0.81)		1.67 (1.26–2.21)		3.16 (2.08–4.80)	
Health indicators						
BMI (Ref: normal)		0.127		0.005		0.004
Underweight	0.78 (0.57–1.06)		1.41 (0.95–2.09)		1.38 (0.84–2.29)	
Overweight	1.08 (0.98–1.19)		1.02 (0.90–1.16)		1.16 (0.99–1.37)	
Obese	1.03 (0.94–1.14)		1.20 (1.06–1.37)		1.34 (1.13–1.58)	
Health status (Ref: excellent)		<0.0001		<0.0001		<0.0001
Very good	0.47 (0.43–0.52)		2.07 (1.77–2.42)		1.99 (1.64–2.42)	
Good	0.34 (0.31–0.38)		3.09 (2.63–3.64)		3.09 (2.52–3.79)	
Fair	0.18 (0.16–0.21)		6.54 (5.36–7.98)		5.53 (4.29–7.13)	
Poor	0.07 (0.05–0.09)		14.83 (9.99–21.99)		10.32 (6.48–16.43)	

HS, High school; Ref, reference group.

**Table 3:** Survey-weighted ordered logistic regression analysis of United States remote-eligible adults by anxiety frequency, anxiety disorder, and anxiety medication usage post COVID-19.

Variable	Anxiety frequency [aOR (95% CI)]	*p*-value	Anxiety disorder [aOR (95% CI)]	*p*-value	Anxiety medication [aOR (95% CI)]	*p*-value
Occupation [remote-eligible] (ref: others)	0.92 (0.86–0.99)	0.028	0.92 (0.82–1.03)	0.168	0.93 (0.82–1.05)	0.228
Sociodemographic characteristics						
Sex (ref: female)		<0.0001		<0.0001		<0.0001
Male	1.87 (1.76–1.99)		0.38 (0.35–0.42)		0.37 (0.33–0.41)	
Age group (yrs) (ref: 18–24)		<0.0001		<0.0001		<0.0001
25–34	1.31 (1.16–1.49)		0.90 (0.75–1.09)		1.00 (0.81–1.23)	
35–44	1.75 (1.53–1.99)		0.74 (0.62–0.88)		0.90 (0.72–1.11)	
45–54	2.64 (2.31–3.02)		0.46 (0.38–0.57)		0.67 (0.54–0.85)	
55–64	3.50 (3.05–4.02)		0.37 (0.30–0.46)		0.68 (0.55–0.86)	
65 and older	5.44 (4.66–6.35)		0.25 (0.20–0.32)		0.51 (0.39–0.66)	
Race/ethnicity (ref: Hispanic)		<0.0001		<0.0001		<0.0001
NH White	0.52 (0.47–0.57)		2.66 (2.26–3.14)		3.03 (2.47–3.71)	
NH Black	0.99 (0.86–1.13)		1.06 (0.85–1.33)		1.06 (0.81–1.39)	
NH Asian	1.29 (1.10–1.51)		0.58 (0.43–0.79)		0.48 (0.32–0.70)	
Others	0.61 (0.46–0.80)		1.99 (1.46–2.71)		1.89 (1.28–2.78)	
Insurance status (ref: uninsured)		0.992		0.002		<0.0001
Insured	1.00 (0.88–1.14)		1.35 (1.11–1.64)		2.16 (1.62–2.87)	
Marital status (ref: not married)		<0.0001		<0.0001		0.0002
Married	1.26 (1.18–1.35)		0.71 (0.64–0.79)		0.81 (0.73–0.90)	
Education (ref: <high school)		<0.0001		<0.0001		<0.0001
High school	0.70 (0.59–0.83)		1.30 (0.99–1.71)		2.09 (1.44–3.03)	
Some college/AA	0.52 (0.44–0.62)		1.54 (1.19–1.99)		2.34 (1.63–3.37)	
Bachelor’s degree	0.42 (0.36–0.50)		1.57 (1.18–2.10)		2.74 (1.87–4.01)	
Graduate degree	0.36 (0.30–0.44)		1.89 (1.41–2.55)		2.99 (2.04–4.37)	
Health indicators						
BMI (ref: normal)		<0.0001		0.077		0.043
Underweight	0.84 (0.62–1.14)		1.59 (1.08–2.33)		1.28 (0.78–2.08)	
Overweight	1.19 (1.10–1.29)		0.97 (0.85–1.10)		1.11 (0.96–1.29)	
Obese	1.26 (1.17–1.37)		1.03 (0.91–1.18)		1.22 (1.06–1.40)	
Health status (ref: excellent)		<0.0001		<0.0001		<0.0001
Very good	0.48 (0.44–0.52)		1.97 (1.69–2.29)		1.83 (1.54–2.17)	
Good	0.36 (0.32–0.39)		2.95 (2.54–3.44)		2.70 (2.26–3.23)	
Fair	0.19 (0.17–1.23)		5.65 (4.60–6.93)		4.01 (3.17–5.08)	
Poor	0.07 (0.05–0.10)		11.60 (7.77–17.31)		7.44 (4.75–11.64)	

HS, High school; Ref, reference group.

### Depression-related mental health outcomes

The adjusted regression models for depression frequency, depression disorder, and depression medication use, respectively, show a significant association with sex, age, race, marital status, education, and health status (Table 2). Male adults were significantly at higher odds of reporting more frequent depression (OR = 1.66; 95% CI: 1.55–1.78), but lower odds of being diagnosed with depression disorder (58%) and using depression medication (64%) (*p* < 0.0001). While depression frequency strongly increases with age, and adults aged 65 years and older had nearly 3 times higher odds of reporting more frequent depression (OR: 2.92; 95% CI: 2.47–3.46), depression disorder, and medication use progressively decrease with age. Adults aged 65 years and older had 58% lower odds of reporting being diagnosed with depression disorder, while those aged 55–64 years (OR: 0.76; 95% CI: 0.59–0.98) and those aged 65 years and older (OR: 0.70; 95% CI: 0.53–0.93) were significantly at lower odds of using depression medication. The non-Hispanic White, non-Hispanic Black, and “Other” race had 44.5, 16.4, and 41.8% lower odds of reporting more frequent depression, but higher odds of being diagnosed with depression disorder [non-Hispanic White: (OR: 2.38; 95% CI: 2.04–2.78); Other: (OR: 2.20; 95% CI: 1.59–3.06)], and using depression medication [non-Hispanic White: (OR: 2.84; 95% CI: 2.29–3.54); Other: (OR: 1.85; 95% CI: 1.19–2.88)] (*p* < 0.0001). Contrary to this, the non-Hispanic Asians were at lower odds of being diagnosed with depression disorder (OR: 0.50; 95% CI: 0.36–0.69) and using depression medication (OR: 0.42; 95% CI: 0.27–0.65) (*p* < 0.0001).

Interestingly, married remote-eligible adults had significantly higher odds of reporting more frequent depression (55%), but lower odds of being diagnosed with depression disorder (33%) and using depression medication (32%) (*p* < 0.0001). Educational attainment was significantly associated with depression frequency, depression disorder, and depression medication use (*p* < 0.0001). Remote-eligible adults holding higher education (some college, bachelor’s, and graduate degrees) were significantly at progressively lower odds of reporting more frequent depression, but higher odds of being diagnosed with depression disorder [some college: (OR: 1.47; 95% CI: 1.15–1.88), bachelor’s: (OR: 1.51; 95% CI: 1.15–1.97), and graduate degrees: (OR: 1.67; 95% CI: 1.26–2.21)]. For depression medication use, remote-eligible adults with higher education beyond <high school showed progressively significantly higher odds of using depression medication. While insurance status showed no significant association with depression frequency, insured remote-eligible adults had 22% higher odds of being diagnosed with depression disorder and 88% higher odds of using depression medication. Obese remote-eligible adults were significantly at 20% higher odds of being diagnosed with depression disorder, and 34% higher odds of using depression medication. Remote-eligible adults who rated their health as poor were at lower odds of reporting more frequent depression (OR: 0.07; 95% CI: 0.05–0.09), but interestingly, at higher odds of being diagnosed with depression disorder (OR: 14.83; 95% CI: 9.99–21.99) and using depression medication (OR: 10.32; 95% CI: 6.48–16.43).

#### Anxiety-related mental health outcomes

The adjusted regression models for anxiety frequency, anxiety disorder, and anxiety medication use, respectively, show a significant association with sex, age, race, marital status, education, and health status (Table 3). Similar to depression health outcomes, male adults were significantly at higher odds of reporting more frequent anxiety (OR = 1.87; 95% CI: 1.76–1.99), but substantially lower odds of being diagnosed with anxiety disorder (61.6%) and using anxiety medication (62.7%) (*p* < 0.0001). While anxiety frequency strongly increases with age, and adults aged 65 years and older had over 440% higher odds of reporting more frequent anxiety (OR: 5.44; 95% CI: 4.66–6.35), anxiety disorder and medication use progressively decrease with age. Adults aged 65 years and older had 75% lower odds of reporting being diagnosed with anxiety disorder (OR: 0.25; 95% CI: 0.20–0.32) and 49% of using anxiety medication (OR: 0.51; 95% CI: 0.39–0.66), respectively. The non-Hispanic White and “Other” race had 48 and 39% lower odds of reporting more frequent anxiety, but higher odds of being diagnosed with anxiety disorder [non-Hispanic White: (OR: 2.66; 95% CI: 2.26–3.14); Other: (OR: 1.99; 95% CI: 1.46–2.71)], and using anxiety medication [non-Hispanic White: (OR: 3.03; 95% CI: 2.47–3.71); Other: (OR: 1.89; 95% CI: 1.28–2.78)] (*p* < 0.0001). Contrary to this, the non-Hispanic Asians had 29% higher odds of reporting more frequent anxiety, but lower odds of being diagnosed with anxiety disorder (OR: 0.58; 95% CI: 0.43–0.79) and using anxiety medication (OR: 0.48; 95% CI: 0.32–0.70) (*p* < 0.0001).-Hispanic White and “Other” race had 48 and 39% lower odds of reporting more frequent anxiety, but higher odds of being diagnosed with anxiety disorder [non-Hispanic White: (OR: 2.66; 95% CI: 2.26–3.14); Other: (OR: 1.99; 95% CI: 1.46–2.71)], and using anxiety medication [non-Hispanic White: (OR: 3.03; 95% CI: 2.47–3.71); Other: (OR: 1.89; 95% CI: 1.28–2.78)] (*p* < 0.0001). Contrary to this, the non-Hispanic Asians had 29% higher odds of reporting more frequent anxiety, but lower odds of being diagnosed with anxiety disorder (OR: 0.58; 95% CI: 0.43–0.79) and using anxiety medication (OR: 0.48; 95% CI: 0.32–0.70) (*p* < 0.0001).

Surprisingly, married adults had significantly higher odds of reporting more frequent anxiety (26%), but lower odds of being diagnosed with anxiety disorder (29%) and using anxiety medication (19%). Educational attainment was significantly associated with anxiety frequency, anxiety disorder, and medication use (*p* < 0.0001). Holding higher education progressively lowers the odds of reporting more frequent anxiety, with remote-eligible adults with a graduate degree at 64% lower odds and those with a bachelor’s degree at 58% lower odds of reporting more frequent anxiety. For anxiety disorder, remote-eligible adults with higher education beyond high school had significantly higher odds of being diagnosed with anxiety disorder and using anxiety medication. While insurance status showed no significant association with anxiety frequency, insured remote-eligible working adults had 35% higher odds of being diagnosed with anxiety disorder and 116% higher odds of using anxiety medication. Overweight (OR 1.192; 95% CI: 1.10–1.29) and obese (OR: 1.26; 95% CI: 1.17–1.37) remote-eligible working adults were significantly at higher odds of reporting anxiety more frequently; only obese remote-eligible adults were at higher odds (22%) of using anxiety medication. Remote-eligible working adults who rated their health as poor were at lower odds of reporting more frequent anxiety (OR: 0.07; 95% CI: 0.05–0.10), but interestingly, at higher odds of being diagnosed with anxiety disorder and using anxiety medication. The odds of being diagnosed with anxiety disorder and using anxiety medication increases as health status deteriorate to poor health, and remote-eligible adults with poorer self-rated health were 11.6 times at higher odds of being diagnosed with anxiety disorder (OR: 11.60; 95% CI: 7.77–17.31), and 7.44 times at higher odds of using anxiety medication (OR: 7.44; 95% CI: 4.75–11.64), which reflects worsening health.

## Discussion

This study assessed the prevalence and associations of six mental health outcomes with sociodemographic and health indicators among remote-eligible working adults in the post-COVID-19 era. While the COVID-19 pandemic led to a significant shift in office workplace arrangements, with working remotely transitioning from a marginal practice to an initial widespread necessity, as a public health measure during the COVID-19 outbreak, full remote work arrangements have become institutionalized in many sectors. After adjusting for all potential covariates in the regression analysis, a clear difference was observed between the reported depression and anxiety frequency, the diagnosed disorders for depression and anxiety, which is the recognition of mental health outcomes, and the medication used for treating depression and anxiety. This difference varies across workers’ sociodemographic characteristics.

### Depression and anxiety-mental health outcomes

#### Sex

Even when male adults had 66 and 87% higher cumulative odds of reporting frequent depression and anxiety, respectively, they were substantially less likely to experience depression disorder, anxiety disorder, use depression medication, or anxiety medication, a direction different from prior studies, which reflects the longstanding gender disparities in mental health. Baños & Miragall (17) found that traditional criteria for diagnosing depression often overlook male-specific symptom expression, leading to systematic under-recognition, while Shi et al. (18) highlight that male depression is mostly hidden or misclassified due to gendered barriers to self-reporting and help-seeking. Consistent with these, prior studies have also reported that gender functions not only as a biological factor but also as a social construct that shapes individuals’ exposure to stressors, access to resources, and coping mechanisms (19, 20). However, the nature of remote work environments, including reduced face-to-face interaction with co-workers, smaller male social networks, and heightened economic or occupational pressures, may have influenced the direction of our findings, making them differ from prior studies.

#### Age

While depression disorder, anxiety disorder, anxiety medication use, and depression medication use progressively decrease with age, with adults aged 55–64 years, and 65 years and older at the lowest odds, depression frequency and anxiety frequency increase with age, a mixed pattern compared to prior studies. Some previous studies found that depression disorder prevalence is higher among younger adults (≥18 years) (21) and younger United States. adults aged 18–25 years are at a 63% increase of depression disorder (22). While others reported that depression symptoms and diagnosed depression and anxiety disorders rise with age, particularly among middle-aged (45–59 years) and older adults (23, 24), and adults aged 55 and older are more likely to use psychotropic medications (25). These inconsistencies could be due to important methodological differences, including variation in age range across studies, as most prior studies, including Twenge et al. (22) and Salk et al. (21), included adolescents in their analyses. This differs from our study, which focused on adults aged 18 + years in specific occupational sectors. However, considering our finding that adults aged 65 years and older are at higher odds of experiencing frequent depression and anxiety, and given the projected aging of the United States workforce, with adults aged 55 years and older expected to comprise nearly one-quarter of the labor force by 2030 through 2050, the need for age-targeted mental health interventions remains essential. Also, continual monitoring of mental health patterns, particularly among younger and older remote-eligible adults, is crucial, especially as chronic illness, cumulative stress exposure, and social isolation may increase mental health vulnerability among the aged 65 and older remote-eligible adults.

#### Marital status

Similar to the findings for age, married adults were at higher odds of reporting frequent depression and frequent anxiety, but lower odds of being diagnosed with depression disorder, anxiety disorder, using anxiety medication, and depression medication, a pattern different from typical traditional epidemiological findings that identify marriage as a protective factor for mental health. Studies show that unmarried adults report higher levels of depressive and anxiety symptoms and use antidepressant or anxiolytic medications (psychotropic medications) more (26–28). While emotional and practical support from family members can buffer against stress, alleviate depression and anxiety symptoms, and promote psychological well-being across the lifespan (29–31), marriage may also coincide with caregiving burdens or interpersonal stressors contributing to anxiety and depressive symptoms. A large household study found that depression is relatively common among married adults (prevalence: 14.4%), with women showing substantially higher rates than men (32). The identified key predictors were multiple marriages, poor relationship quality with one’s spouse, chronic medical comorbidities, and longer conjugal duration (7–12 years) among women, while hard or physically demanding occupations significantly increased risk among men. Additionally, reports also show that marital distress driven by poor communication, relational conflict, unmet expectations, and emotional withdrawal is strongly associated with increased symptoms of depression and anxiety among married individuals (33, 34). Our noticeable difference may also potentially be influenced by increased household demands, work–family conflict, chronic medical conditions of spouse, hormonal changes due to life events such as pregnancy, postpartum transitions or menopause, caregiving responsibilities, financial strain, power imbalances and resentment due to large education or income gap between partners, marriage duration, and individual coping strategies that married remote-eligible working adults encounter, which can disproportionately elevate their psychological distress.

#### Race/Ethnicity

Noticeable racial disparities in both the depression and anxiety outcomes were observed. The non-Hispanic Black adults were at lower odds of reporting frequent depression, a similar and concerning trend to a prior study (35). Bailey et al. (35) reported that African Americans experiencing socioeconomic stress are less likely to report psychological symptoms, which contributes to the under-recognition and delayed diagnosis of depression among this race. This could explain our findings and the need for future studies. Although the non-Hispanic White and “Other” races were at lower odds of reporting frequent depression and anxiety, they were significantly at higher odds of being diagnosed with depression disorder, anxiety disorder, and using depression medication and anxiety medication. In contrast, the non-Hispanic Asians were at higher odds of reporting frequent anxiety, but lower odds of being diagnosed with anxiety disorder, depression disorder, using anxiety medication, and depression medication, similar to patterns in prior studies. Prior population-based studies consistently found the highest depression prevalence among NH White adults (36, 37) and the lowest levels of anxiety and depression among NH Asian adults (38). These observed racial disparities patterns may broadly reflect differences in anxiety and depression disorders diagnosis and treatment rather than differences in symptom frequency burden, which may be shaped by (i) cultural variations in symptom expression, (ii) mental health literacy, and (iii) help-seeking behaviors, as well as (iv) structural inequities including discrimination, limited access to appropriate care, and differential diagnostic pathways (39–42).

#### Insurance status and educational attainment

Insured adults were at higher odds of depression disorder, anxiety disorder, using depression medication, using anxiety medication, and educational attainment showed a mixed pattern across the six assessed mental health outcomes. Adults holding higher education, particularly bachelor’s and graduate degrees, were at higher odds of being diagnosed with depression disorder, anxiety disorder, using depression medication, and anxiety medication, even though they were at lower odds of reporting more frequent depression and anxiety. This contradicts prior studies, including Burger, Becker, & Schoon (43), that reported that individuals with higher levels of education generally experience better mental health outcomes, particularly in young adulthood, which was attributed to increased access to resources, greater job stability, and enhanced social capital. For instance, Goodwin et al. (44) reported that anxiety frequency increases more rapidly among adults with some college education; Kondirolli and Sunder (45) found that each additional year of education was associated with a 9–11% reduction in depression and anxiety symptoms. Although little is known about the association between educational attainment and anxiety medication use, and depression medication use, as our study is the first to assess this mental health outcomes, the possible reasons for remote-eligible adults with higher education to be at higher odds of depression disorder, anxiety disorder, use depression medication, and use anxiety medication may be due to occupational pressures, including work overload, increased job demands, or professional stress, as working remotely within the selected six SOC codes requires higher education and professional expertise.

#### Health indicator

BMI showed mixed findings, with obese adults at higher odds of anxiety frequency, using anxiety medication, depression disorder, and depression medication use, a consistent finding with some prior studies. The association between BMI and mental health is complex and inconsistent, and the association is often influenced by various factors, including perceived stigma, differential healthcare access, variation in help-seeking behavior, and comorbid physical health conditions (46–49). Surprisingly, adults who rated their health as poor were at lower odds of reporting frequent depression, frequent anxiety, but they are at higher odds of being diagnosed with depression disorder, anxiety disorder, using depression medication, and anxiety medication, which reflects worsening health. This seemingly protective association between poorer self-rated health and lower depression and anxiety frequency may reflect treatment effects and diagnostic processes, as people with poorer health have greater clinical contact and are more likely to be diagnosed with depression and anxiety disorders, and receive depression and anxiety medication factors which can reduce the frequency of reported depression and anxiety symptoms while simultaneously increasing their likelihood of documented depression disorder and medication use. However, the findings highlight the intertwined nature of physical and psychological well-being of remote-eligible working adults, with general health status emerging as a stronger predictor of depression and anxiety disorders and treatment.

### Strengths and limitations

The cross-sectional design of the study poses a significant challenge in establishing a clear temporal relationship between remote-eligibility and mental health outcomes, and precludes causal inference, as the analyzed data were self-reported and may be subject to recall bias and not clinical evaluation. It remains unclear whether the “remote-eligible” environment serves as a stressor that exacerbates mental health issues, or if individuals with pre-existing vulnerabilities are disproportionately self-selecting into these occupations for their perceived flexibility. This potential for reverse causality warrants a more cautious interpretation of the findings. Also, remote work status might have been misclassified due to the use of SOC codes to infer remote work and the lack of accountability for unmeasured hybrid arrangements, which may introduce residual confounding. As a result, the observed mental health outcomes could reflect not only the remote work environment itself but also unmeasured job-related stressors, including cognitive load and performance pressure inherent to participants’ professional roles. Also, the findings need to be interpreted with caution, as some of the self-reported depression medications might have been prescribed for anxiety, and vice versa. Despite these limitations, the study has notable strengths. A significant strength of this manuscript is its reliance on the 2023 NHIS. By utilizing this large-scale, nationally representative dataset, the authors provide timely and highly generalizable evidence on the mental health landscape of the United States workforce in the post-pandemic era. The use of such a robust, high-quality data source lends substantial weight to the population-level disparities identified in the study and ensures that the findings are applicable to a broad demographic context. The comprehensive adjustment for sociodemographic and health indicators helps strengthen the internal validity of our findings by reducing confounding bias. Also, the simultaneous assessment of multiple mental health-related outcomes, including symptom frequency, disorder diagnosis, and medication use for both depression and anxiety, provides an integrated view of mental health burden and care.

## Conclusion

There are substantial mismatches between the six assessed mental health outcomes among United States. remote-eligible working adults. There are persistent gaps in mental health, with married males, older adults, particularly aged 65 years and older, and non-Hispanic Asians more likely to experience frequent depression and anxiety. In contrast, non-Hispanic White and “Other” races are more likely to experience anxiety disorder, depression disorder, use anxiety medication, and depression medication. Higher educational attainment and being married did not protect remote-eligible adults against adverse mental health outcomes. As remote work continues to expand, with the projected aging workforce, the need for targeted mental health screening, gender-sensitive and culturally competent care, and equitable and accessible mental health care services is crucial for this workforce.

## Data Availability

The dataset is publicly available and accessible at no charge at: https://www.cdc.gov/nchs/nhis/documentation/?CDC_AAref_Val=https://www.cdc.gov/nchs/nhis/data-questionnaires-documentation.htm.
